# Supplementary material for: Lack of the Histone Deacetylase SIRT1 Leads to Protection against Endoplasmic Reticulum Stress through the Upregulation of Heat Shock Proteins
Source: Int J Mol Sci. 2024 Mar 1;25(5):2856. doi: 10.3390/ijms25052856 (PMC10932345; doi:10.3390/ijms25052856)
Supplement: Supplementary file 1 [file ijms-25-02856-s001.zip › ijms-2852173-Supplementary.pdf]

SUPPLEMENTAL DATA

Lack of the Histone Deacetylase SIRT1 leads to Protection against Endoplasmic Reticulum Stress through the Upregulation of Heat Shock Proteins

Jessica Latorre[1], Nuria de Vera[1], Tomàs Santalucía[2], Rafel Balada[1], Anna Marazuela-Duque[3], Alejandro Vaquero[3], Anna M. Planas[1,4], Valérie Petegnief[1,4*].

Material included: 2 figures and a table.

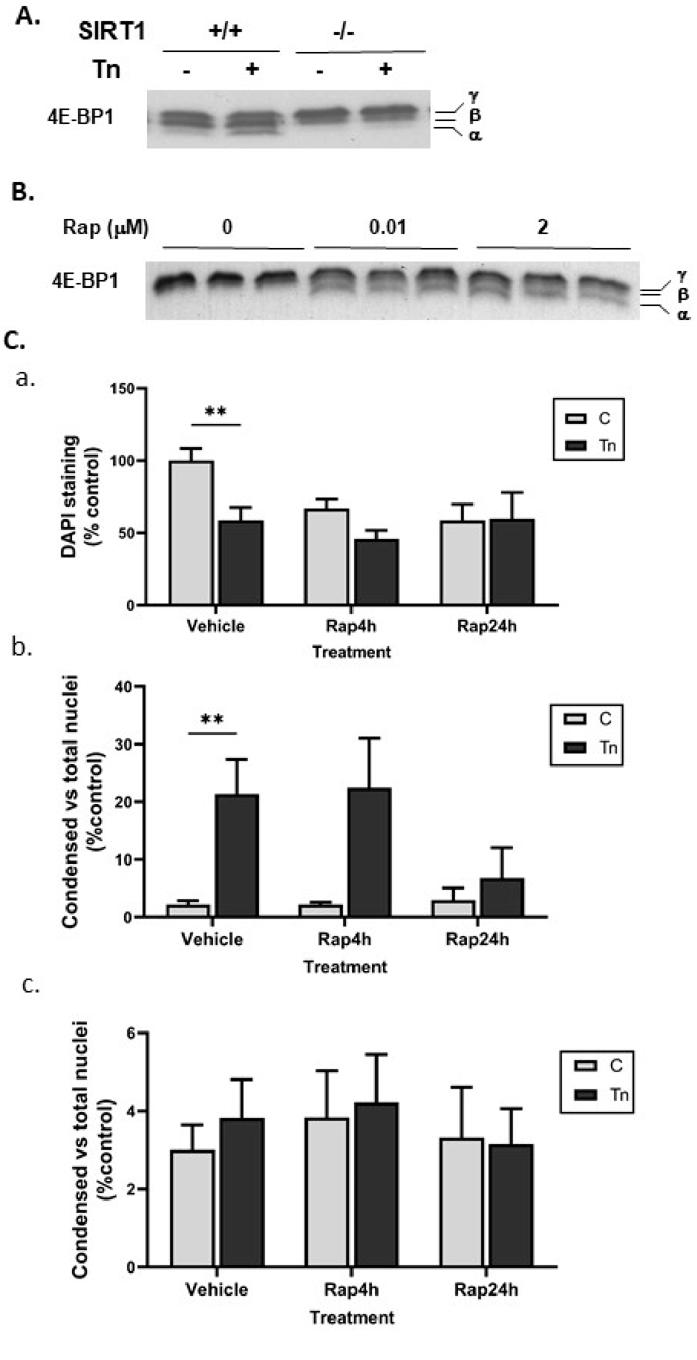

Figure S1. mTOR was overactivated in SIRT1-deficient cells and was not responsible for increased resistance to ER stress.

A. Western blot of 4E-BP1 in control and tunicamycin-treated cells showing the different phosphorylated forms of 4E-BP1 in SIRT1 KO cells and SIRT1 wt cells. Note the absence of the α band: the less phosphorylated band of 4E-BP1 and the high level of the hyperphosphorylated γ band in KO cells. B.

Western blot of 4E-BP1 at 24h after the treatment of SIRT1−/− cells with rapamycin (Rap). C. Cells were treated for 4 or 24h with rapamycin 2 μM and exposed to tunicamycin. Nuclei stained with DAPI were counted and expressed as % of respective control. DAPI staining showed that treatment with 2 μM Rap for 4h partly reduced tunicamycin toxicity and at 24h, Rap abolished tunicamycin toxicity (FigS1Ca, n=3). DAPI staining of healthy and condensed nuclei (FigS1Cb, n=3) and lactate dehydrogenase activity in culture medium (FigS1Cc, n=3) were measured 24h after the tunicamycin addition. Results are mean ± SEM, **P<0.01.

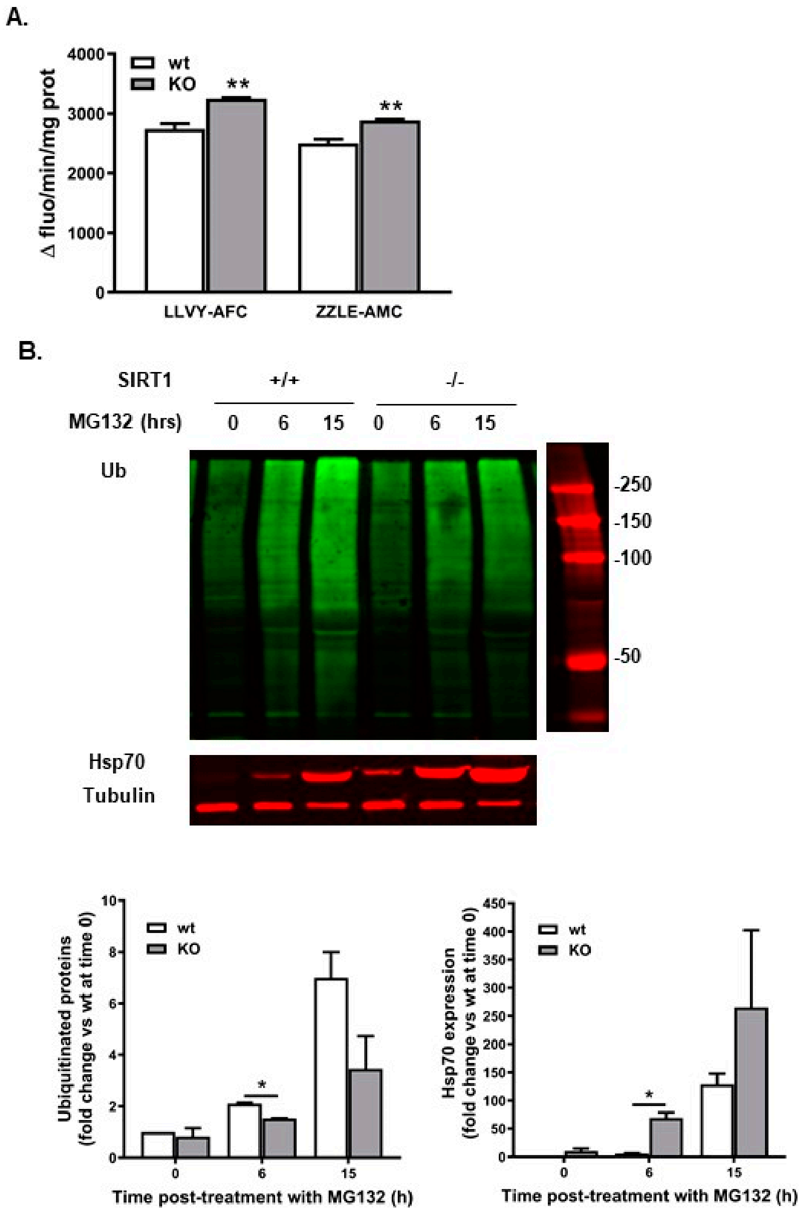

Figure S2. Proteasome activity was higher in SIRT1 KO cells and the proteasome blockade differently affected the accumulation of ubiquitinated proteins and induction of Hsp70 in SIRT1+/+ and SIRT1−/− cells

A. Chymotrypsine and peptidyl-glutamyl-peptidase enzymatic activity were monitored by the cleavage of LLVY-AFC and ZZLE-AMC fluorescent peptides respectively in control wt and SIRT1 KO cells. Results are expressed as mean ± SEM (n=3), **P<0.01. B. Western blot showing the expression of

ubiquitinated proteins and Hsp70 after addition of MG132. Ubiquitination of high molecular weight proteins (75-250 kDa) was quantified (left graph). Results are expressed as mean ± SEM, n=2, *P<0.05.

Table S1. RT-PCR, ChIP primers and shRNA sequences.

**RT-PCR primers**	**Location**	**Forward sequence (5'-3')**	**Reverse sequence (5'-3')**
**Hsp70**		GGCTGATCGGCCGCAAGTT	GGAAGGGCCAGTGCTTCAT
**L14**		GGCTTTAGTGGATGGACCCT	ATTGATATCCGCCTTCTCCC
**Socs-3**		GCGAGAAGATTCCGCTGGTA	CGTTGACAGTCTTCCGACAAAG
**ChIP primers**			
**Hsp70 promoter**[1]	+7/-143	AGGGAGGCGGGGAAGCTCC	GTCTGGTGACCTGCTCGCCG
**Socs3 promoter**[2]	-1247/-1039	CCCCCAACTTCTCATTCACA	TACATGAGGACCTCGGAGTG
**Negative control**	-1164/-1001	AGAAGAAATGGGGCTGGACG	TCCGGAGTGCTGGAATCCTA
**shRNA**		**Sequence (5'-3')**	
**155**		TGTGCTTCCTCTTGAACTC	
**156**		TAGAGATGACCATCTCCAG	
**160**		AACCTACTTAGATTAAATG	
**NS**		ATCTCGCTTGGGCGAGAGTAAG	

References:

1. Sasi BK, Sonawane PJ, Gupta V, Sahu BS, Mahapatra NR. Coordinated transcriptional regulation of Hspa1a gene by multiple transcription factors: crucial roles for HSF-1, NF-Y, NF-kappaB, and CREB. J Mol Biol. 2014;426(1):116–35.

2. Hutchins AP, Diez D, Takahashi Y, Ahmad S, Jauch R, Tremblay ML, et al. Distinct transcriptional regulatory modules underlie STAT3's cell type-independent and cell type-specific functions. Nucleic Acids Res. 2013;41(4):2155–70.
